# Transgenerational programming of longevity through E(z)-mediated histone H3K27 trimethylation in *Drosophila*

**DOI:** 10.18632/aging.101107

**Published:** 2016-11-25

**Authors:** Brian Xia, Ed Gerstin, Dustin E. Schones, Wendong Huang, J. Steven de Belle

**Affiliations:** ^1^ Canyon Crest Academy, San Diego, CA 92130, USA; ^2^ Department of Diabetes Complications and Metabolism, City of Hope, Duarte, CA 91010, USA; ^3^ Department of Biology, Dart Neuroscience LLC, San Diego, CA 92131, USA

**Keywords:** transgenerational inheritance, nutritional programming, longevity, H3K27me3, E(z)

## Abstract

Transgenerational effects on health and development of early-life nutrition have gained increased attention recently. However, the underlying mechanisms of transgenerational transmission are only starting to emerge, with epigenetics as perhaps the most important mechanism. We recently reported the first animal model to study transgenerational programming of longevity after early-life dietary manipulations, enabling investigations to identify underlying epigenetic mechanisms. We report here that post-eclosion dietary manipulation (PDM) with a low-protein (LP) diet upregulates the protein level of E(z), an H3K27 specific methyltransferase, leading to higher levels of H3K27 trimethylation (H3K27me3). This PDM-mediated change in H3K27me3 corresponded with a shortened longevity of F0 flies as well as their F2 offspring. Specific RNAi-mediated post-eclosion knockdown of E(z) or pharmacological inhibition of its enzymatic function with EPZ-6438 in the F0 parents improved longevity while rendering H3K27me3 low across generations. Importantly, addition of EPZ-6438 to the LP diet fully alleviated the longevity-reducing effect of the LP PDM, supporting the increased level of E(z)-dependent H3K27me3 as the primary cause and immediate early-life period as the critical time to program longevity through epigenetic regulation. These observations establish E(z)-mediated H3K27me3 as one epigenetic mechanism underlying nutritional programming of longevity and support the use of EPZ-6438 to extend lifespan.

## INTRODUCTION

Accumulating evidence has supported early-life nutritional programing in the long-term health of an individual and their offspring [1–3]. In fact, the association between early-life nutrition and adult health and disease has been recognized as a cornerstone of public health nutrition programs globally. The World Health Organization, for example, has published global targets and a comprehensive implementation plan for the nutrition of mothers, infants, and young children, aiming to alleviate the double burden of malnutrition in children, starting from the earliest stages of development [4]. Such nutrition-mediated programming effects have often guided early-life nutritional interventions and been shown to be heritable across generations, supporting “early-life nutrition” and “transgenerational inheritance” as key elements of developmental programming [5]. Nevertheless, the underlying mechanisms are poorly defined, with epigenetics as perhaps the most important mechanism, as diet and nutrition can directly alter epigenetic modifications and consequently affect gene expression without altering the underlying DNA sequence [5–7]. Epigenetic modifications including DNA methylation, histone modifications and non-coding RNA-based mechanisms are long-lasting and even heritable. Such epigenetic “marks” established by early-life nutrition may therefore influence subsequent health later in adult life and even across generations [1, 5–10].

Transgenerational inheritance of nutritional programming of metabolic status and longevity has been recently demonstrated in flies, supporting the use of this simple but genetically tractable system for studying the epigenetic inheritance of nutritional programming [11, 12]. Importantly, this model offers multiple advantages, in particular to identify and characterize the epigenetic mechanisms underlying nutritional programming of longevity across generations. First, the relatively shorter rearing period and lifespan of *Drosophila* facilitate longevity experiments over multiple generations in a reasonable time scale [12]. Second, various dietary manipulations and well-conserved (e.g., insulin/IGF, TOR and sirtuin) signaling pathways have been described and characterized for studies of longevity in flies [11, 13–25], all of which have been necessary and critical for rapid identification and characterization of epigenetic mechanisms. Third, all major epigenetic mechanisms (e.g., DNA methylation, histone modifications and non-coding RNA) are present in this fly model system [26], although DNA methylation in flies appears to be different from other eukaryotic organisms and present only at a very low level in adults [27, 28]. Clear evidence has demonstrated that histone modifications [13, 29–32] and at least two microRNAs [33, 34] participate in the regulation of longevity. Finally, recent demonstration of nutritional programming of metabolism and longevity up to the F2 generation [11, 12] has revealed the post-eclosion adult stage to be suitable for assaying the epigenetic mechanisms underlying transgenerational programming of longevity in *Drosophila*.

H3K27me3 is a repressive methylation mark on histone H3 established by the polycomb repressive complex (PRC2) through its core catalytic subunit, the H3K27-specific methyltransferase encoded by the E(z) gene in flies [35] and EZH2 in mammals [36]. PRC2 is highly evolutionary conserved even in unicellular alga *Chlamydomonas reinhardtii* and budding yeast *Cryptococcus neoformans* [35–38]. This is significant, as diet and nutrition affects longevity across diverse single-celled, invertebrate and vertebrate animals [16]. Interestingly, EZH2 may be deacetylated and negatively regulated by Sirt1 [39, 40], an evolutionarily conserved nutrition sensor and well-characterized longevity gene [14, 41, 42], suggesting that E(z)/EZH2 may function downstream of Sirt1 to regulate nutrition-mediated longevity.

Importantly, existing evidence supports a role of the E(z)/EZH2-containing PRC2 for longevity across species. A common PRC2 signature marked by EZH2 and SUZ12 (another core component of PRC2) has been reported for aging-associated genes, suggestive of PRC2 as a potentially common mechanism of aging in humans [43]. Consistently, polycomb repression appears to be associated with healthy aging in humans [44], and replicative senescence of stem cells, an *in vitro* aging-related process [45, 46]. The E(z)-containing PRC2 has also been implicated in longevity regulation in *Drosophila*, as heterozygous mutations of E(z) increase longevity while reducing H3K27me3 levels in adults [31]. Interestingly, E(z)-mediated H3K27me3 is required for paternal transmission of obesity through reprogramming of metabolic genes in *Drosophila* [47], suggesting that H3K27me3 may be involved with transgenerational reprogramming. Finally, UTX-1 (an H3K27-specific histone demethylase) has been shown to regulate lifespan, and transgenerational epigenetic inheritance of longevity has been reported for H3K4me3 (H3K4 trimethylation) in *C. elegans* [48–52]. Significantly, H3K27me3 and H3K4me3 are the frequent antagonistic partners found on the bivalent chromatin domains that be implicated in aging and aging-related diseases in humans [53, 54].

In this study, we examined whether E(z)/EZH2-dependent H3K27me3 may be one epigenetic mechanism underlying transgenerational inheritance of nutrition-programmed longevity. As EZH2 has been actively pursued as a therapeutic target for various cancers [55], its inhibitors were also examined for their potential effect on longevity.

## RESULTS

### Transgenerational longevity decrease and H3K27me3 upregulation after the LP PDM in the F0 parents

The LP diet (with high sugar content) was employed, as high-sugar diets have been known to cause nutritional programming of metabolic status and aging-related diseases including diabetes, diabetic cardiomyopathy and diminished memory [11, 18, 23, 24, 56]. Virgin male flies were used, as longevity experiments would last a shorter time with males, and the transgenerational programming effects on longevity appear to be independent of gender, mating and reproduction after the LP PDM [12]. Newly-born F0 flies were subjected to a 7-day PDM with the LP diet. The longevity and western analyses were performed with these treated F0 males and their F2 male flies while being maintained on the CD food throughout their whole developmental and adult lives (i.e., without any additional exposure to the LP food across the F0–F2 generations). The F1 generation was not assayed, as the intergenerational transmission to F1 reflects both parental effects and transgenerational programming [12].

**Figure 1:**
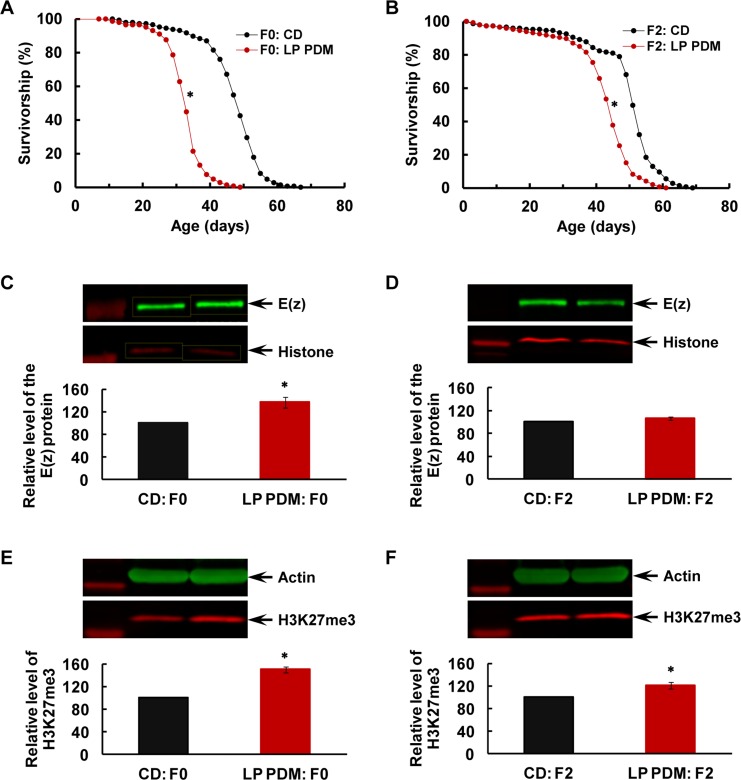
Transgenerational longevity decrease and H3K27me3 upregulation after the LP PDM in the F0 parents (**A-B**) Survival curves for virgin males, (**C-D**) E(z) protein level (with sample westerns shown above the quantification in the same order of respective lanes; same for all the following figures with western analyses), and (**E-F**) H3K27me3 level in the F0 parents (**A**, **C** and **E**) and the F2 flies (**B**, **D** and **F**). After 7-day PDM of the F0 parents with the LP diet (in red) or control diet (CD; in black), all the F0 flies and their F1 and F2 offspring were maintained on the CD at all times. N=145-147 for longevity analyses, and N=4 for western analyses of E(z) and H3K27me3. The asterisk (*) indicates a significant difference from control (see main text for specific P values).

Longevity was reduced in the F0 and their F2 males (Figure 1A–B; P<0.0001 for both comparisons, Mantel-Cox test) after the 7-day LP PDM. The E(z) protein level was upregulated in the F0 parents (Figure 1C; P=0.02 for LP PDM vs. CD, one sample T-test) after the LP PDM, and the upregulation was not observed in the F2 generation (Figure 1D; P=0.11). In contrast, the H3K27me3 level was increased in both F0 and F2 flies (Figure 1E–F; P=0.002 and 0.03). These observations suggest that early-life dietary changes may disturb E(z)-mediated H3K27me3 through misregulation of the E(z) protein, and consequently nutrition-induced H3K27me3 dysfunction may be transmitted across generations and underlie transgenerational inheritance of nutritional programming of longevity.

### Transgenerational longevity extension and H3K27me3 downregulation after specific post-eclosion RNAi-mediated knockdown (KD) of the E(z) gene in the F0 parents

Two independent, specific RNAi lines, E(z) TRiP #33659 and #27793, were crossed to an HS-Gal4 line (see Figure S1 for the targeted regions of both RNAi transgenes and the detailed experimental design involving the UAS/Gal4 gene expression system). Virgin males and females were collected from their progeny (designated as F0 parents for post-eclosion RNAi KD), carrying both the HS-Gal4 and RNAi trans-genes, and subjected to 2x 60-min heat shock (from 25°C to 37°C; see Methods for details) per day for 7 days. Upon heat shock, the RNAi transgenes were induced in whole flies and then would recognize and bind to their respective targeted regions in the E(z) gene for KD as specific post-eclosion RNAi-mediated KD of E(z) in the F0 parents. The longevity and western analyses were performed with these treated F0 males and their white-eyed F2 males while being maintained on the CD food at 25°C throughout their whole developmental and adult lives (i.e., without any additional heat shock across the F0–F2 generations).

**Figure 2:**
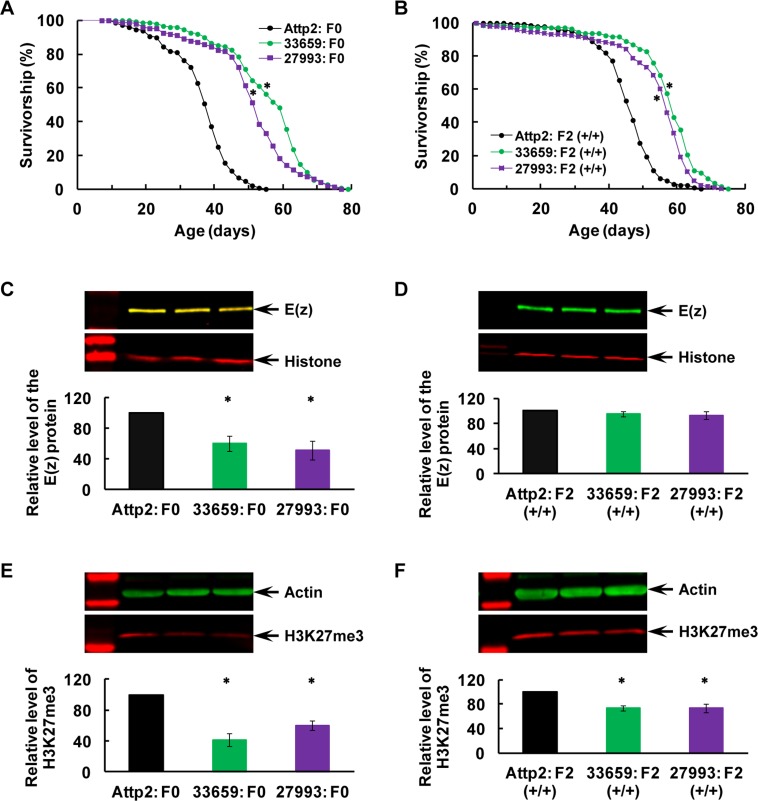
Transgenerational longevity extension and H3K27me3 downregulation after specific post-eclosion RNAi-mediated KD of the E(z) gene in the F0 parents (**A-B**) Survival curves for virgin males, (**C-D**) E(z) protein level, and (**E-F**) H3K27me3 level in the F0 parents (**A**, **C** and **E**) and their F2 offspring (**B**, **D** and **F**). All the flies were raised on CD at all times. Specific RNAi-mediated KD of E(z) was induced twice per day via heat shock (25 → 37°C for 1 hour) for 7 days immediately after eclosion. Two independent lines were used (33659, in green; or 27993 in purple), with their parental line as the control (Attp2, in black). Genotypes: Attp2: F0 — *HS-Gal4; Attp2 / +; +*, 33659: F0 — *HS-Gal4; 33659 RNAi / +; +*, 27793: F0 — *HS-Gal4; 27793 RNAi / +; +*, Attp2: F2 (+/+) — *+; + / +; +*, 33659: F2 (+/+) — *+; + / +; +*, 27793: F2 (+/+) — *+; + / +; +*. N=145-149 for longevity analyses, and N=4 for western analyses of E(z) and H3K27me3. The asterisk (*) indicates a significant difference from control (see Table S1 for detailed analyses and specific P values).

For the F1 generation, about one in four flies from the control cross (HS-Gal4 x Attp2) or one in sixteen from the RNAi cross (HS-Gal4 × 33659 or 27793) had a white-eyed phenotype, indicating the absence of either RNAi or HS-Gal4 transgene. White-eyed F1 virgin males and females were collected and mated to generate the F2 offspring flies. Among these F2 flies, white-eyed males (i.e., +; + / +; +, carrying no RNAi or HS-Gal4 transgene; designated as Attp2: F2 (+/+), 33659: F2 or 27793: F2 (+/+) in Figure 2) were used for longevity and western blotting experiments.

In support of the specificity of RNAi-mediated E(z) KD, longevity, E(z) protein and H3K27me3 levels were all found to be normal in the F0 males without heat shock (Figure S2), suggesting that there was no substantial leaky expression from the HS-Gal4 transgene and thus no non-specific effect on longevity or H3K27me3 was induced.

Longevity was extended in the F0 parents after early-adult-specific E(z) KD, and the extension was also observed in their F2 generation (Figure 2A–B; P≤0.0001 for all comparisons, Mantel-Cox test; see Table S1 for detailed analyses). Western blotting confirmed that the E(z) protein level was greatly reduced via specific RNAi-mediated KD with either RNAi transgene in the F0 flies (Figure 2C; P=0.01, one-way ANOVA, followed with post-hoc Fisher's LSD tests with ɑ=0.05; Table S1), but was back to normal in the F2 generation (Figure 2D; P=0.48, one-way ANOVA; Table S1). In contrast, H3K27me3 was greatly downregulated in the F0 parents because of the E(z) KD (Figure 2E; P=0.0003, ANOVA, followed with Fisher's LSD tests with ɑ=0.05; Table S1), and this downregulation was also seen in their F2 offspring (Figure 2F; P=0.003, ANOVA, followed with Fisher's LSD tests with ɑ=0.05; Table S1). These observations support that the E(z)-mediated H3K27me3 changes in the F0 parents may be transmitted across generations and therefore underlie transgenerational programming of longevity.

### Transgenerational longevity extension and H3K27me3 downregulation after post-eclosion inhibition of the E(z) enzymatic function in the F0 parents

EPZ-6438 was employed for inhibiting E(z) catalytic function, as E(z)/EZH2 is highly evolutionary conserved [35–38]. This inhibitor has been shown to be highly EZH2 selective, with 35-fold selectivity over EZH1 (the other mammalian homologue of E(z)) and >4500 fold over all other histone methyltransferases [57]. EZH2 has been actively targeted for various cancers, with EPZ-6438 as the most advanced (in phase I or II clinical trials for non-Hodgkin's lymphoma, B-cell lymphoma, synovial sarcoma, renal, soft tissue sarcoma, breast and mesothelioma cancers) among all currently active cancer drug programs [55, 57]. The drug also shows the highest brain penetration among all inhibitors tested [58]. Importantly, EPZ-6438 is orally available [57], allowing easy delivery through feeding dissolved in vehicle or food media.

**Figure 3:**
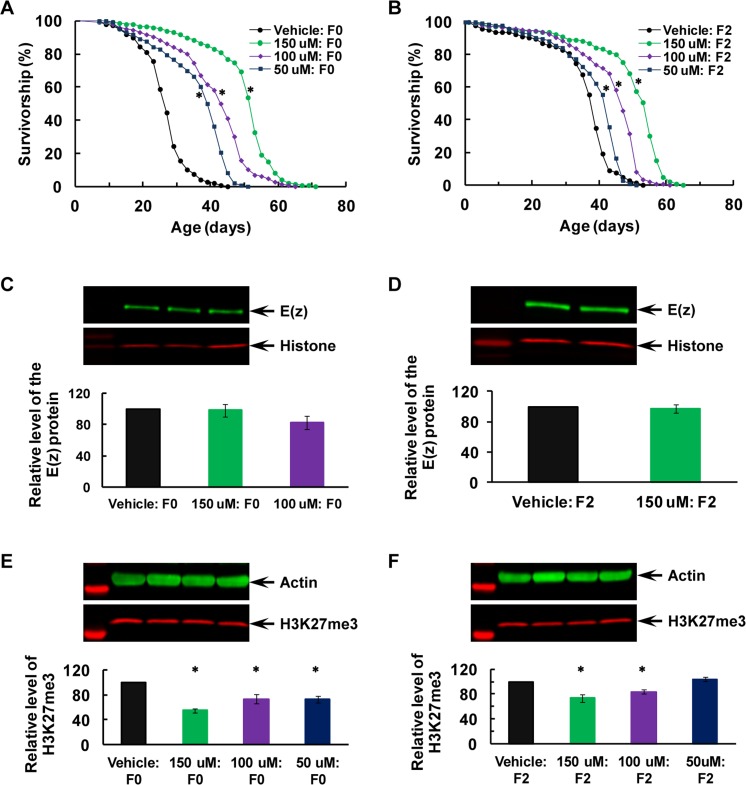
Transgenerational longevity extension and H3K27me3 downregulation after post-eclosion inhibition of the E(z) enzymatic function in the F0 parents (**A-B**) Survival curves for virgin males, (**C-D**) E(z) protein level, and (**E-F**) H3K27me3 level in the F0 parents (**A**, **C** and **E**) and their F2 offspring (**B**, **D** and **F**). All the flies were raised on CD at all times after EPZ-6438 feeding dissolved in 5% sucrose as vehicle. Inhibition of the E(z) methyltransferase activity was induced for 7 days following eclosion in a dose-dependent manner. The doses of 100, 200 and 400 μM were first used to determine an effective dose at 100 μM through western analyses of H3K27me3 (see Figure S3 for details). Then 50 μM (in blue), 100 μM (in purple) or 150 μM (in green) EPZ-6438, or vehicle (in black) were used for subsequence longevity and western analyses. N=145-147 for longevity analyses, and N=4 for western analyses of E(z) and H3K27me3. The asterisk (*) indicates a significant difference from control (see Table S2 for detailed analyses and specific P values).

The newly-eclosed virgin males and females were fed with vehicle (5% sucrose) or various doses of EPZ-6438 for 7 days before being maintained on the CD food throughout their developmental and whole adult lives (i.e., without any additional drug feeding) across the F0–F2 generations). Longevity of the males, the E(z) protein and H3K27me3 levels were assayed in the F0 parents and their F2 offspring (Figure 3). Longevity was extended in the F0 flies in a dose-dependent manner (Figure 3A; P<0.0001 for all comparisons, Mantel-Cox test; see Table S2 for detailed analyses), and the extension was heritable to their F2 generation and still dose-dependent (Figure 3B; P<0.0001 for all comparisons, Mantel-Cox test; Table S2). The E(z) protein level was not affected by EPZ-6438 feeding in the F0 parents or F2 flies (Figure 3C–D; P=0.28, one-way ANOVA for F0, and P=0.77 for “150μM” vs. vehicle, one sample T-test for F2; Table S2), suggesting that the inhibitor has no effect on E(z) translation.

H3K27me3 was greatly reduced in the F0 parents (Figure 3E; P=0.0002, one-way ANOVA, followed with Fisher's LSD tests with ɑ=0.05; Table S2) because of the inhibition of E(z) function by EPZ-6438, and this decrease was also seen in their F2 generation for the two higher doses, 150 and 100μM (Figure 3F; P=0.003, one-way ANOVA, followed with Fisher's LSD tests with ɑ=0.05; Table S2). These observations suggest that this phase II EZH2-selective compound also inhibits the catalytic function of E(z), and further support that the E(z)-dependent H3K27me3 changes in the F0 flies may be transmitted to their F2 offspring and thus underlie transgenerational programming of longevity.

### Transgenerational alleviation of the LP-induced longevity decrease after post-eclosion inhibition of the E(z) enzymatic function in F0 parents with EPZ-6438

**Figure 4:**
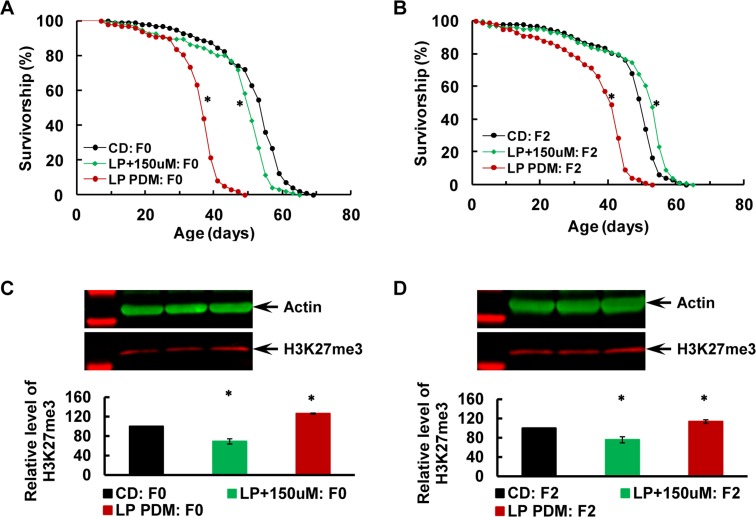
Transgenerational alleviation of the LP-induced longevity decrease after post-eclosion inhibition of the E(z) enzymatic function in F0 parents with EPZ-6438 (**A-B**) Survival curves for virgin males, and (**C-D**) H3K27me3 level in the F0 parents (**A** and **C**) and their F2 offspring (**B** and **D**). The F0 parents were subjected to the 7-day PDM with the LP diet (in red) along or LP diet containing EPZ-6438 at the concentration of 150 μM (LP+150μM; in green) and with CD as the control (in black). N=96-99 for longevity analyses, and N=4 for western analyses of H3K27me3. The asterisk (*) indicates a significant difference from control (see Table S3 for detailed analyses and specific P values).

The newly-eclosed virgin males and females were fed with the LP diet alone, or LP diet with EPZ-6438 added at the concentration of 150μM (LP+150uM) for 7 days, and with CD as the control before assaying for longevity of the males and the H3K27me3 level in the F0 males and their F2 male offspring (Figure 4). Longevity was reduced in the F0 flies subjected to the LP PDM, but nearly fully restored with addition of EPZ-6438 (Figure 4A; P<0.0001 for LP vs. CD or LP+150μM vs. LP, and P<0.0001 for LP+150μM vs. CD; see Table S3 for details). Longevity was also shortened in the F2 flies after the LP PDM of the F0 parents (Figure 4B; P<0.0001 for LP vs. CD; Table S3), but slightly extended after addition of EPZ-6438 to the LP diet (P=0.0003, LP+150uM vs. CD), implying that the alleviation effect of EPZ-6438 may be improved across generations. Western blotting confirmed an upregulation of H3K27me3 after the LP PDM, while addition of EPZ-6438 to the LP diet reduced H3K27me3 below the CD control level (Figure 4C; P<0.0001, one-way ANOVA, followed with Fisher's LSD tests with ɑ=0.005; Table S3), suggesting that the LP-induced H3K27me3 upregulation was inhibited with EPZ-6438. The H3K27me3 level was also higher than the control in the F2 flies after the LP PDM, while lower after addition of EPZ-6438 (Figure 4D; P=0.0004, one-way ANOVA, followed with Fisher's LSD tests with ɑ=0.05; Table S3), suggesting that the LP-induced H3K27me3 upregulation and its inhibition by EPZ-6438 was transmitted to the F2 generation.

Longevity was reduced by the LP PDM while almost fully restored after addition of EPZ-6438 to the LP diet; and in parallel, the LP-induced H3K27me3 upregulation was selectively inhibited (to a level even below the control) by the EZH2-selective inhibitor. Importantly, the EPZ-6438-mediated alleviation effect on longevity decrease and H3K27me3 misregulation was transmitted from the F0 parents to their F2 offspring. These observations clearly demonstrate that the E(z)-dependent H3K27me3 dysregulation may be the primary cause of the LP-induced longevity decrease, and H3K27me3 may underlie the nutritional programming of longevity.

### Early-life period as the critical time to extend longevity via H3K27me3 inhibition

**Figure 5:**
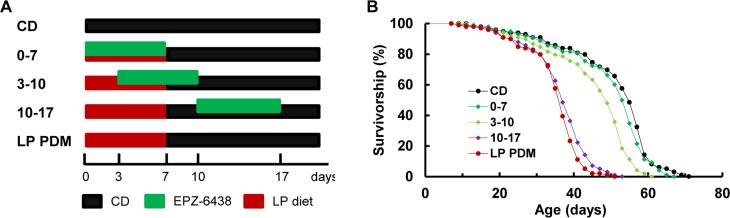
Early-life period as the critical time to extend longevity via H3K27me3 (**A**) Experimental design, and (**B**) survival curves for virgin males. The flies were subjected to one of five dietary and EPZ-6438 manipulations as illustrated in (**A**). N=98-99 for longevity analyses. Statistical analyses and median lifespan were summarized in Table S4.

Longevity was assayed as the flies were subjected to one of five post-eclosion dietary and EPZ-6438 manipulations as illustrated in Figure 5A: 1) CD at all times as control, 2) the LP PDM, 3) the LP PDM with EPZ-6438 added to the LP diet (“0-7”), 4) the LP PDM with EPZ-6438 added to the LP diet from day 4–7 and then to CD from day 8–10 (“3-10”), and 5) the LP PDM with EPZ-6438 added to CD from day 10–17 (“10-17”). Only when EPZ-6438 was administrated simultaneously as the LP PDM, were the LP-induced longevity decrease fully alleviated (Figure 5B; P=0.10 for “0-7” vs. CD; see Table S4 for details). The EPZ-6438-dependent alleviation effect became gradually lessened as the inhibitor was administrated later in adult life (P<0.0001 for “3-10” vs. LP and P=0.04 for “10-17” vs. LP; Table S4). Noticeably, the alleviation effect was essentially negated when the drug was administrated 3 days after the LP PDM (with “10-17” and LP groups showing the same median lifespan at 37 days and P=0.04). These observations support the immediate early-life period as the critical time to extend longevity through an epigenetic therapy.

## DISCUSSION

Our observations demonstrate that E(z)-dependent H3K27me3 activity may underlie transgenerational inheritance of nutritional programming of longevity, further validating our model system to study nutritional programming of longevity and its cross-generation inheritance [12]. We have also identified an EZH2-selective inhibitor (i.e., EPZ-6438) for extending longevity in a long-lasting and even transgenerational manner. Considering that EPZ-6438 is generally safe as a phase II candidate cancer drug [57], translational studies may be initiated for other aging-related diseases. Importantly, EPZ-6438 showed greater efficacy in alleviating the LP-induced reduction of longevity when administrated earlier in adult life, supporting early-life interventions for improving health span and potentially delaying aging-related diseases.

Our data convincingly demonstrate that E(z)-mediated H3K27me3 activity may play a critical role in the general health of an organism and therefore its longevity, consistent with recent reports that aging-associated genes share a common PRC2 signature marked by EZH2 and SUZ12, suggestive of PRC2 as a potentially common mechanism of aging in humans [43]. The LP PDM was found to upregulate the E(z) protein level in the F0 parents, and this upregulation was not seen in the F2 flies. Nevertheless, the resulting increase of E(z)-dependent H3K27me3 was observed in the F0 parents and their F2 offspring, supporting the idea that early-life dietary changes may disturb E(z)-mediated H3K27me3 activity through misregulation of the E(z) protein, and therefore diet-induced H3K27me3 changes may underlie transgenerational inheritance of nutritional programming of longevity through its cross-generation transmission (Figure 1). This conclusion was greatly strengthened with the next two experiments -RNAi-mediated early-life specific knockdown of E(z) only in the F0 parents improved longevity while reducing H3K27me3 activity across generations (Figure 2), and early-adult life inhibition of E(z) enzymatic function with EPZ-6438 also improved longevity while rendering the H3K27me3 level low across generations (Figure 3). Importantly, the E(z) protein level was normal in the F2 generation after its KD in the F0 parents (Figure 2D) and was not affected by EPZ-6438 even in the F0 parents (Figure 3C), while H3K27me3 was reduced in both situations, arguing that the RNAi-mediated KD or EPZ-6438-induced inhibition may be specific. The specificity was further supported when normal longevity, E(z) and H3K27me3 levels were observed without heat shock to induce RNAi transgenes (Figure S2), and as EPZ-6438 is highly EZH2 selective and generally safe [57]. Most importantly, addition of EPZ-6438 to the LP diet greatly alleviated the longevity-reducing effect of the LP PDM while inhibiting LP-induced H3K27me3 upregulation across the F0 to F2 generations, convincingly demonstrating that the LP-induced longevity reduction was primarily caused by an increased level of E(z)-dependent H3K27me3 (Figure 4). This conclusion was further supported by the time-dependent loss of the EPZ-6438-induced alleviation effect (Figure 5). Therefore, our results have revealed E(z)-mediated H3K27me3 as one epigenetic mechanism underlying nutritional program-ming of longevity, and identified the EZH2 inhibitors (e.g., EPZ-6438) as promising molecules to extend health span, even across generations.

Identification of the EZH2 inhibitor EPZ-6438 for improving longevity (across generations) opens up the possibility to evaluate its potential efficacy in various aging-related diseases. As a known nutrition sensor and NAD-dependent deacetylase, Sirt1 has been extensively characterized for longevity regulation, and its activators have been shown to extend longevity and confer broad health benefits against aging-related diseases, including diabetes, heart disease, neurodegeneration and cancers [14, 41, 42]. Interestingly, EZH2 is deacetylated and negatively regulated by Sirt1 [39, 40], suggesting that E(z)/EZH2 may function downstream of Sirt1 to regulate nutrition-mediated longevity programming. Considering that addition of EPZ-6438 to the LP diet alleviated the LP-induced longevity decrease, it will be interesting to evaluate EPZ-6438's potential efficacy in alleviating LP-induced type II diabetes, cardiovascular disease and memory impairment [11, 18, 24]. Impor-tantly, H3K27me3 is a highly conserved epigenetic modification [35–38] and EZH2-containing PRC2 has been found to mark aging-associated genes in humans [43]. Similar mechanisms may be expected to function during aging in humans, supporting the idea that these observations may be transferrable to humans. Consider-ing that EPZ-6438 is generally safe as a phase II candidate cancer drug [57], translational studies may be initiated quickly for other disease indications. A “super-drug” capable of simultaneous prevention of multiple aging-related diseases in a long-lasting and even transgenerational manner would greatly increase our opportunity to cure many diseases while reducing the disease burden and cost.

Accumulating studies have established a strong link between early-life nutrition and adult health and disease, leading to the formation of the Developmental Origins of Health and Disease (DOHaD) approach for studying many aging-related diseases including cardiovascular disease, obesity, type 2 diabetes and metabolic disturbances, some forms of cancer, and mental illnesses [1, 10, 59–62]. The DOHaD research of 3 decades contests that “*it is no longer possible for adult medicine to ignore the developmental phase of life*” [61] and “*it is therefore questionable whether the efficacy of interventions that currently form the mainstay of NCD* (non-communicable disease) *risk reduction in adults could be greater than that obtained from interventions made earlier in life*” [63]. However, the direct evidence is still lacking to support superior efficacy of early-life interventions. Here with the alleviation effect on the LP-induced longevity decrease as the general health benefits conferred by EPZ-6438, we have generated one example of such evidence for EZH2 inhibitors (Figure 5). The alleviation effect was found to be greatest, intermediate or very mild when EPZ-6438 was administrated within the first 7 days, from day 3–10 or from day 10-17 after eclosion, respectively. In fact, the alleviation effect was even seen in the F2 generation when the inhibitor was delivered within the first 7 days after eclosion (Figure 4B), while essentially negated when the drug was administrated 3 days after the LP diet (Figure 5B). Our data therefore support the DOHaD approach for studying aging-related disease in *Drosophila* and the use of the develop-mentally appropriate time period for interventions. Beside the epigenetic therapies which are expected to be long-lasting and even inheritable under certain circumstances, it would be worth exploring whether non-epigenetic therapies have improved efficacy for early-life interventions.

For our post-eclosion treatments, the germ cells (future gametes) from the F0 flies (males and females) would also be directly exposed to the treatment influence, while those from the F1 females would not [12]. In this regard, we were treating both young adult F0 flies and F0 germ cells. As the F2 flies were generated from F1 germ cells never exposed to these treatments, any displayed programming effects must result from transgenerational inheritance or transmission, likely through the germline. Thus the programming effects of longevity observed in F0 flies would not necessarily reflect those transmitted to the F2 flies. Consistent with this idea, longevity was slightly shortened in the F0 flies, while mildly extended in the F2 flies, as compared with the CD control, after addition of EPZ-6438 to the LP diet (Figure 4). A cautionary note therefore would be to use clearly defined developmental stages such as early embryonic or pupal period, when applying the DOHaD approach to *Drosophila* studies.

Our results also serve as the first proof-of-concept for transgenerational longevity-improving epigenetic therapy with EPZ-6438 for extending health span, and potentially for aging-related diseases. This important concept was supported by transgenerational extension of longevity via post-eclosion feeding of EPZ-6438, and transgenerational alleviation of the LP-induced longevity decrease after adding the inhibitor to the LP diet (Figures 3–4). When combined with personalized medicine (i.e., therapy decisions tailored to individual patients based on genetic risk information and molecular characterization; cf. [64–66]) and the DOHaD approach, longevity-improving epigenetic therapies may have huge implications for drug discovery and health care. First, such therapeutic interventions delivered at an early developmentally-appropriate time period may be very effective for simultaneous prevention of multiple aging-related diseases in adults and even across generations (as discussed above). In addition, one important trend for drug discovery is the ongoing shift from single-target-oriented molecules to network- or biological system-active compounds and to “epi-drugs” [67–69]. Epigenetic targets typically regulate a large number of genes through epigenetic modifications [70], and individual inhibitors or activators may achieve network-active purpose on their own. Finally, such knowledge can also be combined with personalized medicine and the DOHaD approach to promote appropriate risk reduction interventions in early life, and motivate healthier choices and meaningful behavior changes in adults.

Our experiments have demonstrated that E(z)-dependent H3K27me3 may underlie transgenerational inheritance of nutritional programming of longevity, and the EZH2-selective inhibitors (e.g., EPZ-6438) may be used to extend longevity in a long-lasting and trans-generational manner. These observations are of significance in identifying the first epigenetic mechanism underlying nutritional programming of longevity, supporting the DOHaD approach for studying aging-related diseases, and providing the first proof-of-concept for a transgenerational epigenetic therapy with an EZH2 inhibitor to extend health span.

## METHODS

### Flies

Wild-type isogenic w^1118^ strain (stock #5905, Bloomington Stock Center) was used throughout the study. For early-adult specific knockdown of E(z), two independent RNAi stocks, E(z) #33659 and #27793 (TRiP collection), and a heat-shock GAL4 (HS-Gal4) line were used. All the flies were maintained in Forma incubators with controlled temperature (25°C) and humidity (40%) on a 12:12 light-dark cycle (with light on at 8am).

### Diet

The control diet (CD) is a food medium routinely used in the laboratory, containing ~8.5% protein and ~76.5% carbohydrate. The “LP” (Low Protein) diet contains much less protein (~3.3%) while much more carbohydrate (~90.5%), and has been shown to program longevity and abolish learning and memory across generations [12, 56]. The food recipes, along with the calorie, protein and carbohydrate information for CD and LP media have been described earlier [12].

### Antibodies

The following primary antibodies were used for western blotting: mouse monoclonal anti-EZH2 (PCRP-EZH2-1B3, Developmental Studies Hybridoma Bank) for detecting and quantifying the E(z) protein, with mouse monoclonal anti-beta actin antibody (ab8224, Abcam) or rabbit polyclonal anti-Histone H3 antibody (ab1791, Abcam) as loading control; or rabbit polyclonal anti-H3K27me3 (07-449, EMD Millipore) for H3K27me3, with above-mentioned anti-beta actin antibody as loading control. IRDye 800CW goat anti-mouse IgG (926-32210, LI-COR) and IRDye 680RD goat anti-rabbit IgG (926-68071, LI-COR) secondary antibodies were multiplexed for simultaneous 2-color detection with Odyssey CLx Imaging Systems (LI-COR).

### EPZ-6438

The compound (A8221, ApexBio Technology) is a highly selective EZH2 (one of the two mammalian homologues for E(z)) inhibitor, with 35-fold selectivity over EZH1 (the 2^nd^ mammalian homologue of E(z)) and > 4500 fold over all other histone methyltransferases [57]. With EZH2 as a target, there are 7 currently active cancer drug programs for non-Hodgkin's lymphoma, B-cell lymphoma, synovial sarcoma, renal, soft tissue sarcoma, breast and mesothelioma cancers (https://clinicaltrials.gov/). EPZ-6438 is the most advanced among these 7 programs, in phase II for multiple cancers. It is orally available, and thus can be fed to flies. It also shows the highest brain penetration among 5 inhibitors tested [58].

### Post-eclosion dietary manipulation (PDM) of F0 parents

The procedure has been detailed before [12]. Briefly, virgin males and females were collected within 4 hours after eclosion, and then maintained on LP or CD for 7 days. Then, various groups of virgin males were used for longevity and western analyses; and about 100 mated females were split into 4 groups and used for generating the F1 and F2 offspring while being maintained on CD at all times. Similarly, longevity and western analyses were done with their F2 offspring.

### Post-eclosion RNAi-mediated specific knockdown of E(z)

Two independent RNAi transgenes (i.e., #33659 and #27993, all inserted to the “Attp2” insertion site) were used (see Figure S1 for their targeted regions and further information). The heat shock-induced knockdown was achieved with the binary UAS/Gal4 expression system (see Figure S1 for detailed description). Progenies from the HS-Gal4 x UAS-E(z)^RNAi^ cross carried both HS-GAL4 and UAS-E(z)^RNAi^ transgenes. Upon heat shock (25-37°C for 1 hour; see below for details), the GAL protein (yeast transcription factor introduced into *Drosophila*) was induced, and then activated RNAi transgenes by binding to UAS (Upstream Activation Sequence, an enhancer to which GAL4 specifically binds to activate transcription of the targeted gene).

### Heat shock protocol

Virgin males and females (progenies from the HS-Gal4 x UAS-E(z)^RNAi^ cross) were collected within 4 hours of eclosion. Groups of about 100 flies were trapped in foam-stoppered empty plastic vials containing a strip of filter paper to absorb extra moisture. The vials then were placed in a small 37°C incubator for 1 hour to induce the RNAi transgenes. After heat shock, the flies were transferred to the CD vials for 11 hours at 25°C for recovery. Next, the whole cycle of 1-hour heat shock (at 37°C in empty food vials) was repeated after every 11 hours (at 25°C in regular CD food vials) for 7 days. Therefore, the flies were heat shocked twice per day for 7 days after eclosion.

### Post-eclosion drug feeding

EPZ-6438 (A8221, ApexBio Technology) was dissolved in DMSO and then diluted with 5% sucrose solution as vehicle. The virgin males and females were fed with various doses of EPZ-6438 for 7 days after eclosion to inhibit E(z) enzymatic function in the F0 parents only. The doses of 100, 200 and 400μM were first used to determine an effective dose with western blotting at 100uM (data not shown), and then doses of 50, 100 and 150μM were used for longevity and further western analyses. EPZ-6438 was also mixed into the LP diet or CD at a concentration of 150μm for longevity analyses (see Figures 4–5 for more information).

### Protein extraction

The adult flies at various ages, dependent on particular experiments, were collected and frozen with liquid nitrogen. For western blotting with the E(z) protein, nuclear proteins were extracted with a nuclear extraction kit (ab113474, Abcam), following manufacturer's protocol. For H3K27me3, histone proteins were extracted with a histone extract kit (ab113476, Abcam), following manufacturer's protocol except that the lysates were incubated on ice for 2 hours, instead of the recommended 30 minutes. The protein concentration was quantified with Pierce BCA protein assay kit (23225, Thermo Fisher Scientific), following manufacturer's protocol, and Envision multilabel plate reader (PerkinElmer) for absorbance reading at 562nm.

### Longevity assay

All data were collected in a blind and balanced manner, with different groups of flies blind-coded and balanced for various sources of variation, including (1) number of flies in each vial and for each PDM, (2) food level across vials, and (3) light exposure, humidity and temperature by regular rotation through fixed locations in incubators. Then a large number of flies (~ 100 or 150) were used to ensure systematic and sufficient data collection, and reproducibility. Flies were transferred onto new CD vials every 2 days, ensuring that all flies had access to fresh food, and the feeding environment remained fresh and consistent. The date and number of dead flies for each vial were recorded at the time when the flies were being transferred. All dead flies were carefully removed with a spatula. Any fly that accidentally escaped or died would not be considered.

The F0 parent generation was first subjected to the 7-day PDMs, RNAi-mediated knockdown of the E(z) gene or functional inhibition of E(z) enzymatic function with EPZ-6438, while their F2 generation was never exposed to these post-eclosion treatments. Therefore, the longevity data were collected from 7-day-old flies for the F0 parents, and 1-day-old flies for their F2 offspring.

### Western blotting

The protein samples were separated with Criterion TGX stain-free precast gels (567-8033, 10% for quantifying E(z), or 567-8094, 4-20% for H3K27me3), and transferred to nitrocellulose membranes with the Trans-Blot Turbo Transfer system (Bio-Rad), following manufacturer's protocols. The membranes were then blocked in Odyssey blocking buffer in PBS form (927-40000, LI-COR) for 1 hour with gentle shaking at room temperature, incubated with anti-EZH2 (1:500) and anti-histone H3 (1:10000), or anti-H3K27me3 (1:5000) and anti-beta actin (1:10000) antibodies for 1–4 hours or overnight at 4°C, and finally incubated with IRDye 800CW (1:10000) and IRDye 680RD (1:10000) secondary antibodies for 1 hour at room temperature. Both primary antibodies and subsequently both secondary antibodies were always multiplexed for simultaneous incubation, and eventually for parallel 2-color detection. The western signals were scanned and quantified with Odyssey CLx Imaging system (LI-COR).

### Data analysis

All longevity analysis was run through GraphPad Prism. Prism uses the Mantel-Cox test to generate survival curves and compares the survival distributions of two samples to determine the significance of any changes. The median lifespan data were also obtained to calculate the percentage changes of the longevity (see Table S1-4 for details).

The E(z) or H3K27me3 level was first normalized to histone H3 or beta actin as the loading control, and the signals from 4 independent repeats were averaged (n=4) to determine the significance of any changes away from a normalized “100” level (one sample T-test), and confirmed with ANOVA for experiments with 3 or more treatment groups, followed by post-hoc Fisher's LSD (least significant difference) tests.

## SUPPLEMENTARY MATERIAL FIGURES AND TABLES


