# Supplementary material for: Transgenerational programming of longevity through E(z)-mediated histone H3K27 trimethylation in Drosophila
Source: Aging (Albany NY). 2016 Nov 25;8(11):2988–3002. doi: 10.18632/aging.101107 (PMC5191882; doi:10.18632/aging.101107)
Supplement: Supplementary file 1 [file aging-08-2988-s001.pdf]

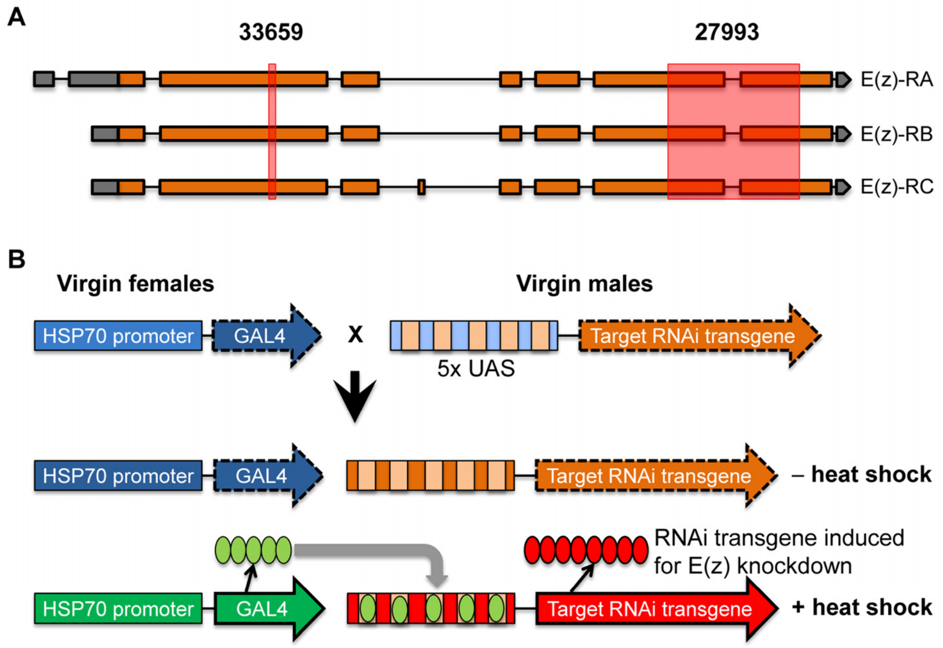

Supplemental Figure S1. Cartoon illustration of specific heat shock-induced RNAi-mediated knockdown of the E(z) gene. (**A**) The intron-exon structure of the E(z) gene and the targeted regions of the RNAi transgenes. The #33659 RNAi transgene was constructed with only 21 bp DNA sequence specific to the E(z) gene, targeting a shared exon (marked in red) by all 3 E(z) isoforms. The #27993 RNAi was constructed with 504 bp DNA sequence, targeting a shared region spanning over 2 shared exons (marked in red) by all 3 E(z) isoforms. (**B**) Illustration of the HS-Gal4/UAS gene expression system. The HS-Gal4 transgene encodes a yeast transcription factor under the control of HSP70 promotor and is introduced into *Drosophila*. The UAS-RNAi transgene is the target RNAi sequence (e.g., 33659 or 27793) under the control of UAS (Upstream Activation Sequence, an enhancer to which GAL4 specifically binds to activate gene transcription) and can be activated upon specific GAL4 binding to UAS. Virgin females, homozygous for HS-Gal4 transgene, are crossed to virgin males, homozygous for the RNAi transgene (top panel). Virgin males and females are collected from their progeny, containing both HS-Gal4 and RNAi transgenes. Without heat shock (– heat shock), both Gal4 and RNAi transgenes are kept silent. With heat shock (+ heat shock), the Gal4 protein is induced, and then binds to UAS so as to induce RNAi transgenes.

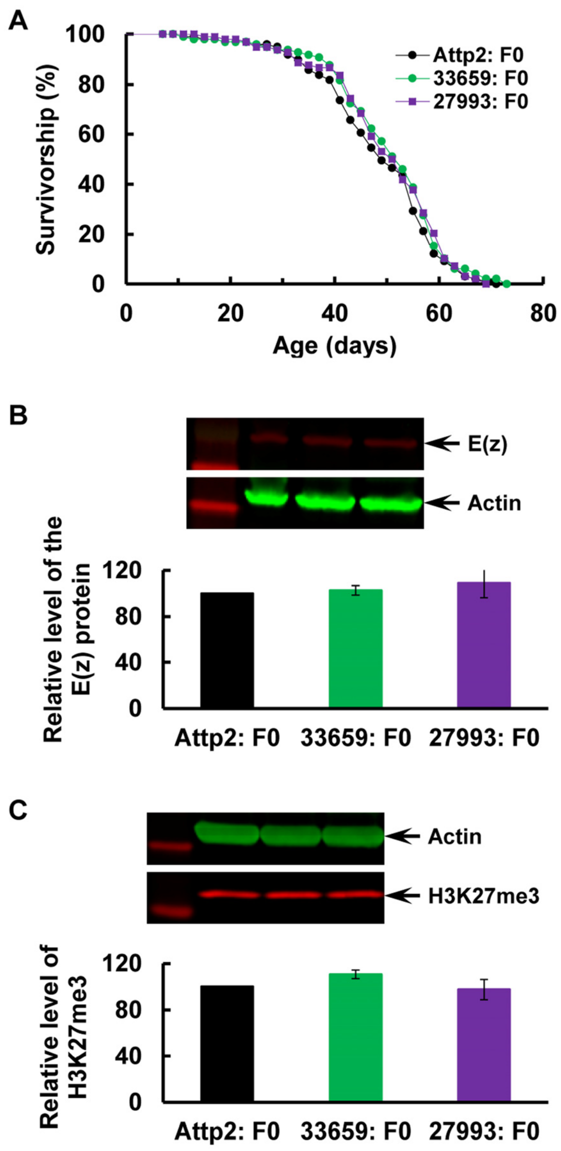

Supplemental Figure S2. **Normal longevity, E(z) and H3K27me3 levels without heat shock.** (**A**) Survival curves for virgin males, (**B**) E(z) protein level, and (**C**) H3K27me3 level in the F0 parents. All the flies were raised on CD at all times. No RNAi-mediated KD of E(z) was induced, as the flies were subjected to the same heat shock procedure twice per day, but without heat shock (25 $\rightarrow$ 25°C for 1 hour in the water bath) for 7 days immediately after eclosion. Both RNAi lines were evaluated (33659, in green; or 27993, in purple), with their parental line as the control (Attp2, in black). Genotypes: Attp2: F0 — *HS-Gal4; Attp2 / +; +*; 33659: F0 — *HS-Gal4; 33659 RNAi / +; +*; 27793: F0 — *HS-Gal4; 27793 RNAi / +; +*. N=98–99 for longevity analyses, and N=2 for western analyses of E(z) and H3K27me3. No significant difference was detected for longevity (P≥0.20 for 33659 or 27793 vs. Attp2; Mantel-Cox test), E(z) protein (P=0.84; one-way ANOVA) or H3K27me3 (P=0.52; one-way ANOVA).

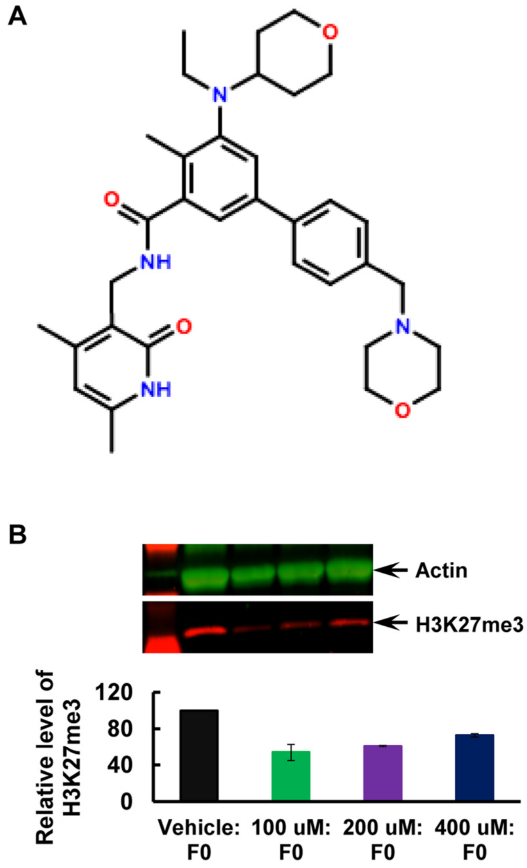

Supplemental Figure S3. **The structure and dose response of EPZ-6438.** (**A**) The structure of EPZ-6438, and (**B**) dose response. The flies were fed with 5% sucrose as vehicle or EPZ-6438 at above-indicated doses for 7 days. Inhibition of the E(z) methyltransferase activity was evaluated through western analyses of the H3K27me3 levels. All three doses produced a significantly lower level of H3K27me3 (P=0.03, one-way ANOVA, followed with Fisher's LSD tests with α=0.05), with 100µM as the most effective dose.

Supplemental Table S1. Statistical analyses for longevity and western data shown in Figure 2.

Data set	Groups	F0 parents: subjected to 7-day RNAi-mediated KD of E(z)				F2 offspring			
**Longevity**		Mantel-cox		Median lifespan		Mantel-cox		Median lifespan	
		Summary	P value	Value	Change	Summary	P value	Value	Change
	Attp2	C	<0.0001 B vs. C	39	–	C	<0.0001 B vs. C	47	–
	33659	A		59	51% ↑	A		59	25% ↑
	27793	B	0.0008 A vs. B	53	36% ↑	B	0.002 A vs. B	57	21% ↑
**E(z) protein**		Normalized value	1-sample T-test	ANOVA	LSD (α=0.05)	Normalized value	1-sample T-test	ANOVA	LSD (α=0.05)
	Attp2	100	–		A	100	–		A
	33659	60±10	0.03	P=0.01	B	95±4	0.28	P=0.48	A
	27793	51±12	0.03		B	93±6	0.32		A
**H3K27me3**		Normalized value	1-sample T-test	ANOVA	LSD (α=0.05)	Normalized value	1-sample T-test	ANOVA	LSD (α=0.05)
	Attp2	100	–		A	100	–		A
	33659	42±9	0.007	P=0.0003	B	73±4	0.005	P=0.003	B
	27793	60±6	0.008		B	73±7	0.03		B

The Mantel-Cox (or log-rank) test was used to compare the survival distribution of "Attp2", "33659", or "27793" flies for the F0 and F2 generations. For the "Summary" column, groups not connected by the same letter were significantly different, with "A" indicating the longest lifespan. The median lifespan data were also obtained to calculate the percentage changes of the longevity, when compared with the "Attp2" control. ↑: significantly improved lifespan. The 33659 RNAi appeared to extend longevity better than the 27793 RNAi transgene. The E(z) or H3K27me3 level was first normalized to histone 3 or beta actin as the loading control, and the signals from 4 independent repeats were averaged (n=4) to determine the significance of any changes away from a normalized "100" level (one sample T-test), and then confirmed with ANOVA, followed with post-hoc Fisher's LSD (least significant difference) tests (α=0.05). For the "LSD" column, groups not connected by the same letter were significantly different, with "A" indicating the highest value.

Supplemental Table S2. Statistical analyses for longevity and western data shown in Figure 3.

**Data set**	**Groups**	**F0 parents: subjected to 7-day inhibition of E(z) function with EPZ-6438**				**F2 offspring**			
**Longevity**		Mantel-cox		Median lifespan		Mantel-cox		Median lifespan	
		Summary	P value	Value	Change	Summary	P value	Value	Change
	Vehicle	D	<0.0001 C vs. D	27	–	D	<0.0001 C vs. D	39	–
	150µM	A		53	96% ↑	A		55	41% ↑
	100µM	B	<0.0001 A vs. B	44	63% ↑	B	<0.0001 A vs. B	47	21% ↑
	50µM	C	<0.0001 B vs. C	40	48% ↑	C	<0.0001 B vs. C	43	10% ↑
**E(z) protein**		Normalized value	1-sample T-test	ANOVA	LSD (α=0.05)	Normalized value	1-sample T-test	ANOVA	LSD (α=0.05)
	Vehicle	100	–		A	100	–		
	150µM	98±8	0.83	P=0.28	A	98±6	0.77	–	
	100µM	85±7	0.13		A	–	–		
	50µM	–	–		–	–	–		
**H3K27me3**		Normalized value	1-sample T-test	ANOVA	LSD (α=0.05)	Normalized value	1-sample T-test	ANOVA	LSD (α=0.05)
	Vehicle	100	–		A	100	–		A
	150µM	55±4	0.001	P=0.0002	C	74±6	0.02	P=0.003	B
	100µM	73±7	0.03		B	84±3	0.01		B
	50µM	73±5	0.01		B	104±3	0.26		A

The Mantel-Cox (or log-rank) test was used to compare the survival distribution of "Vehicle", "150µM", "100µM" or "50µM" flies for the F0 and F2 generations. For the "Summary" column, groups not connected by the same letter were significantly different, with "A" indicating the longest lifespan. The median lifespan data were also obtained to calculate the percentage changes of the longevity, when compared with the "Vehicle" control. ↑: significantly improved lifespan. The E(z) or H3K27me3 level was first normalized to histone 3 or beta actin as the loading control, and the signals from 4 independent repeats were averaged (n=4) to determine the significance of any changes away from a normalized "100" level (one sample T-test), and then confirmed with ANOVA, followed with post-hoc Fisher's LSD (least significant difference) tests (α=0.05). For the "LSD" column, groups not connected by the same letter were significantly different, with "A" indicating the highest value.

Supplemental Table S3. Statistical analyses for longevity and western data shown in Figure 4.

Data set	Groups	F0 parents: subjected to 7-day LP PDM or LP+150μM EPZ-6438				F2 offspring			
		Mantel-cox		Median lifespan		Mantel-cox		Median lifespan	
		Summary	P value	Value	Change	Summary	P value	Value	Change
Longevity	CD	A		55	–	B	0.0003 A vs. B	51	–
	LP+150μM	B	<0.0001 A vs. B	51	8% ↓	A		53	4% ↑
	LP PDM	C	<0.0001 B vs. C	37	33% ↓	C	<0.0001 B vs. C	41	20% ↓
H3K27me3		Normalized value	1-sample T-test	ANOVA	LSD (α=0.05)	Normalized value	1-sample T-test	ANOVA	LSD (α=0.05)
	CD	100	–		B	100	–		B
	LP+150μM	69±5	0.01	P<0.0001	C	76±6	0.03	P=0.0004	C
	LP PDM	126±1	0.0002		A	114±4	0.03		A

The Mantel-Cox (or log-rank) test was used to compare the survival distribution of "CD", "LP+150μM", or "LP PDM" flies for the F0 and F2 generations. For the "Summary" column, groups not connected by the same letter were significantly different, with "A" indicating the longest lifespan. The median lifespan data were also obtained to calculate the percentage changes of the longevity, when compared with the "CD" control. ↓: significantly shortened lifespan; ↑: significantly improved lifespan. The "LP+150μM" group showed mild longevity reduction for the F0 flies, but mild longevity extension for the F2 flies. The H3K27me3 level was first normalized to beta actin as the loading control, and the signals from 4 independent repeats were averaged (n=4) to determine the significance of any changes away from a normalized "100" level (one sample T-test), and then confirmed with ANOVA, followed with post-hoc Fisher's LSD (least significant difference) tests (α=0.05). For the "LSD" column, groups not connected by the same letter were significantly different, with "A" indicating the highest value.

Supplemental Table S4. Statistical analyses for longevity curves shown in Figure 5.

	**Mantel-cox test**		**Median lifespan**	
	Summary	P value	Value	Change
CD	A		55	–
0-7	A	0.10 for 0-7 vs. CD	54	2% ↔
3-10	B	<0.0001 for 3-10 vs. 0-7	49	11% ↓
10-17	C	<0.0001 for 10-17 vs. 3-10	37	33% ↓
LP PDM	D	0.04 for LP PDM vs. 10-17	37	33% ↓

The Mantel-Cox (or log-rank) test was used to compare the survival distribution of "CD", "0-7", "3-10", "10-17" or "LP PDM" flies (F0). For the "Summary" column, groups not connected by the same letter were significantly different, with "A" indicating the longest lifespan. The median lifespan data were also obtained to calculate the percentage changes of the longevity, when compared with the "CD" control. ↓: significantly shortened lifespan; ↔: no significant lifespan change.
